# Sacrifice Few to Save Many: Fire Protective Interlayers in Carbon-Fiber-Reinforced Laminates

**DOI:** 10.1021/acsomega.4c01408

**Published:** 2024-05-22

**Authors:** Weronika Tabaka, Dietmar Meinel, Bernhard Schartel

**Affiliations:** Bundesanstalt für Materialforschung und−prüfung (BAM), Unter den Eichen 87, 12205 Berlin, Germany

## Abstract

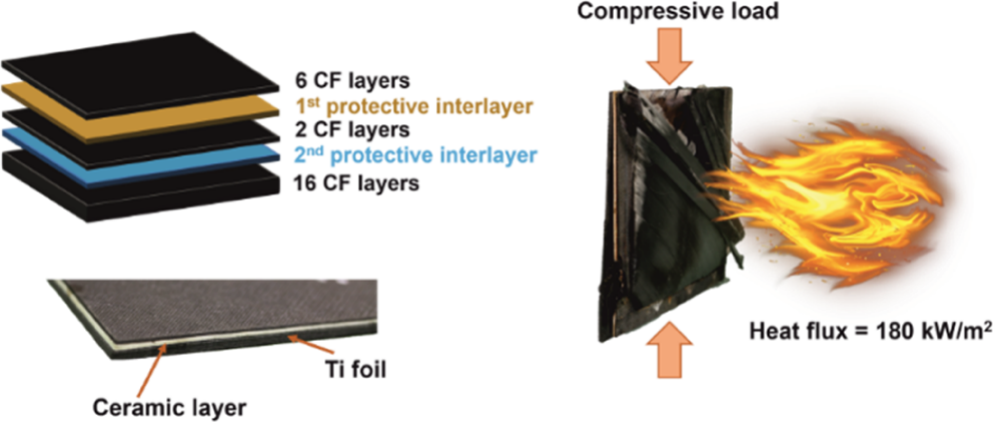

The fire protection of carbon-fiber-reinforced polymer (CFRP) laminates often relies on flame-retardant coatings, but in some applications, their efficacy may diminish upon direct fire exposure due to rapid pyrolysis. This study introduces an innovative approach by integrating protective interlayers within the laminate structure to enhance the fire resistance. Various materials, including ceramic composite WHIPOX, titanium foil, poly(ether imide) (PEI) foil, basalt fibers, rubber mat, and hemp fibers, were selected as protective interlayers. These interlayers were strategically placed within the laminate layout to form a sacrificial barrier, safeguarding the integrity of the composite. Bench-scale fire resistance tests were conducted, where fire (180 kW/m^2^) was applied directly to the one side of the specimen by a burner while a compressive load was applied at the same time. Results indicate significant prolongation of time to failure for CFRP laminates with protective interlayers, which is up to 10 times longer. This innovative approach represents a potential advance in fire protection strategies for CFRP laminates, offering improved resilience against fire-induced structural failure.

## 1 Introduction

Flame-retardant coatings provide thermal insulation against heat and are the most popular fire protection for composites in load-bearing applications.^1−5^ Using coatings offers several advantages, e.g., they are easy to apply to the specimen and help avoid the delamination of carbon fiber layers. The improvement of fire resistance of CFRP laminates with protective coatings has been presented in previous work.^6^ However, direct exposure to fire can drastically reduce the effectiveness of coatings due to almost spontaneous pyrolysis and massive ablation. Furthermore, other drawbacks of coatings have been observed, i.e., additional weight without load-carrying support, weak bonding to the substrate, poor mechanical properties, and weathering issues. Therefore, this research presents an alternative approach to fire protection by incorporating protective interlayers into the carbon-fiber-reinforced polymer (CFRP) laminate structure.

A laminate is a type of composite material in which thin layers are joined together. The individual layers determine the characteristics of laminates; thus, some properties can be predicted and designed before manufacturing by selecting appropriate parameters, such as fiber orientation, ply thickness, stacking sequence, and volume fraction.^7−9^ Fiber-reinforced polymer laminates are widely used in industrial, marine, aerospace, and construction applications. However, due to the flammable polymer-organic matrix, their fire performance is poor. In particular, the load-bearing capacity of composites deteriorates, resulting in failure of the laminate structure.^10−17^

The approach of protective interlayers has been studied before, i.e., by Timme, Christke, and Schuett,^18−20^ but these works focused mainly on the integration of one type of protective layer into a composite. Incorporating two different protective interlayers in the laminate structure improves fire performance through the influence of the different properties of the individual layers and their position in the laminate lay-up.

There are different materials that were already investigated and provided effective protective layers. WHIPOX is a wound, highly porous oxide matrix composite developed by the German Aerospace Center (DLR). It has excellent mechanical and thermal properties and was designed for high-temperature applications in the aerospace and energy sectors. In addition, its porous matrix provides for nonbrittle behavior, which increases the material’s attractiveness in compression tests.^21−23^ The combination of metal and composites is an extensively utilized solution, especially for aerospace applications, to benefit from high strength, lightweight, and fire protection properties. Titanium is particularly intriguing due to its ability to not only reduce heat conductivity and improve fire protection but to significantly enhance fire stability as well.^18,19,24−26^ Poly(ether imide) foil (PEI) is a high-performance thermoplastic with good compatibility to epoxy resin, which expands at high temperatures (above 540 °C) and forms a barrier with lower thermal conductivity than CFRP laminate. In addition, it is a load-carrying layer, which improves the toughness of the composite.^20,27,28^ Growing interest in sustainability has led to the development of “green” composites, which offer environmentally friendly alternatives to conventional materials. Natural fibers, known for their low density and impressive mechanical and physical properties, are not only abundant but also biodegradable. Consequently, they are widely used as reinforcements in composites. Basalt fibers extruded from molten volcanic rock have excellent properties such as high strength and durability, as well as low thermal conductivity (in the range of 0.031–0.038 W/m·K) and a high operating temperature limit (∼700 °C). Moreover, basalt fibers have been investigated as reinforcements in composites, often in combination with other fibers such as carbon or glass.^29−34^ Plant fibers are becoming increasingly preferred over synthetic fibers. The strength and stiffness of hemp (*Cannabis sativa*) fiber makes it a versatile material with a wide range of applications.^35,36^ Although they are characterized by relatively high flammability, they can be modified or combined with, e.g., synthetic fibers or flame retardants to achieve promising fire protection properties.^37−42^

This research investigated six distinct systems of CFRP laminates with five different protective interlayers. The materials chosen as protective interlayers are the commercially available products, used in different applications, aviation, construction, and also natural fibers, which nowadays are very attractive and desired materials. The lay-up configuration was specifically designed so that the foremost section of the laminate, comprising 8 carbon fiber layers (in two parts: 6CF and 2CF) accompanied by 2 protective interlayers, is sacrificed to controlled combustion and thus shields the underlying component, which consists of 16 carbon fiber layers. This study is focused on the fire resistance of CFRP laminates, which is very important for many applications. Fire resistance was tested in a bench-scale setup, where fire was applied directly to the one side of the specimen by a burner while compressive load was applied simultaneously. The bench-scale fire stability test is a viable and effective method to evaluate different fire protection approaches and their influence on the structural integrity of CFRP in fire.^6,43^ The compressive force used in the fire test was 10% of the ultimate failure load of the pure CFRP shell. The study evaluates the impact of interlayer thickness and material type on fire resistance. X-ray computed tomography was employed to analyze the failure modes of residues. Our studies present that the time to failure for CFRP laminates with protective interlayers is significantly prolonged.

## 2 Experimental Section

### 2.1 Materials

The carbon fiber layers (Tenax – E IMS65 E23 24K Aircraft Quality) were supplied by C.Cramer GmbH & Co (Heek, Germany) as an unidirectional carbon fiber fabric ECC UD 134CIM with a weight per unit area of 134 g/m^2^. Epoxy resin (EPIKOTE Resin MGS RIMR 935) and hardener (EPIKURE MGS RIMH 937) were purchased from Hexion Inc. (Columbus, Ohio). Six different materials were used for integrated fire protection interlayers. WHIPOX fiber-reinforced ceramic composite was manufactured by WPX Faserkeramik GmbH (Germany) and has a thickness of 0.5 mm. Titanium foil was supplied by ATI Flat Rolled Products GmbH (Germany) and had a thickness of 125 μm. Ajedium Ultra 1000 poly(ether imide) (PEI) foil is 125 μm thick and was supplied by Solvay (Germany). The rubber mat Pyrostat Uni was purchased from G+H ISOLIERUNG GmbH and is 1.1 mm thick. This rubber band expands during fire and forms a fire-proof barrier. To improve resin flow between the layers and to provide good saturation of the carbon fiber layers, small holes (1 mm) were drilled along the entire length (20 mm distance between holes) of Ti foil, PEI foil, and the rubber mat. The basalt-fiber-woven mat was supplied by Incotelogy Ltd. (Germany) and has a thickness of 100 μm. The 100% hemp natural fiber mat was provided by Polyvlies Franz Beyer (Germany). To improve integration with CF, it was first saturated with epoxy resin by hand-laying supported by vacuum bagging. The final thickness of the natural fiber mat was 2.2 mm.

### 2.2 Manufacturing

**Figure 1:**
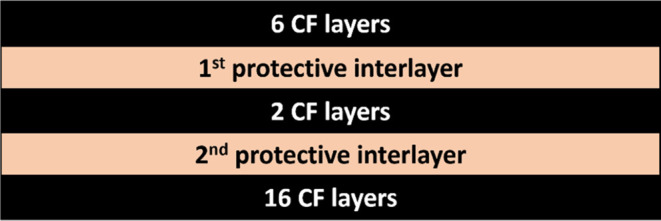
Schematic illustration of the arrangement of protective interlayers in the composite laminate.

The reference specimen was a quasi-isotropic CFRP laminate composed of 24 carbon layers: [±/90/–/0/+/90/0/–/90/+/0]s, prepared by Vacuum-Assisted Resin Transfer Molding (VARTM). Due to the specific viscosity of the epoxy resin and to achieve fully saturated carbon fiber layers, the manufacturing process of CFRP laminates with protective interlayers was divided into 2 steps. First, each of the carbon fiber lay-ups (CF-6L, CF-2L, CF-16L) was prepared using VARTM. The resin was mixed with the hardener in the weight ratio 100:38 and degassed. The mold was 1470 mm wide and 2900 mm long and had a curvature diameter of 4150 mm. The curvature corresponds to the typical shell structure used for an aircraft fuselage and was *R* = 2500, which increases the buckling stability of laminates.^18^ In order to keep a proper resin viscosity, the mold was heated to 30 °C during infusion using a heating mat placed underneath. After 3 h, the temperature was increased to 90 °C to accelerate the cure. To avoid embrittlement of the composite and to cure the resin, the material was postcured for 5 h at 160 °C. The carbon fiber lay-ups were then joined with first and second protective interlayers by a hand lay-up method, supported by vacuum bagging, which ensured to fully infuse the laminate and helped to avoid the resin excess. The schematic view in Figure 1 shows where the interlayers are located in the laminate lay-up. The first part of the laminate, 6 layers of carbon fibers, the first interlayer, 2 layers of carbon fibers, and the second interlayer, is sacrificed and serves as fire protection for the rest of the laminate, 16 carbon fibers. The idea of inserting protective interlayers into the laminate structure was previously investigated by Timme, who concluded that protection from direct flame contact must be provided. Therefore, in this work, the number of carbon fiber layers in the front part was increased to six.^18^ The configuration of each layout is presented in Table 1.

**Table 1:** Structure of CFRP Shells with Incorporated Protective Interlayers; PL: Protective Layer

lay-ups	configuration (+:45°,–:135°)
CF-6L	[+/–/90/–/0/+]
CF-2L	[0/90]
CF-16L	[−/90/+/0/0/+/90/–/0/90/+/0/–/90//–/+]
CF + 1st PL + 2nd PL	[CF-6L]/1st PL/[CF-2L]/2nd PL/[CF-16L]

**Figure 2:**
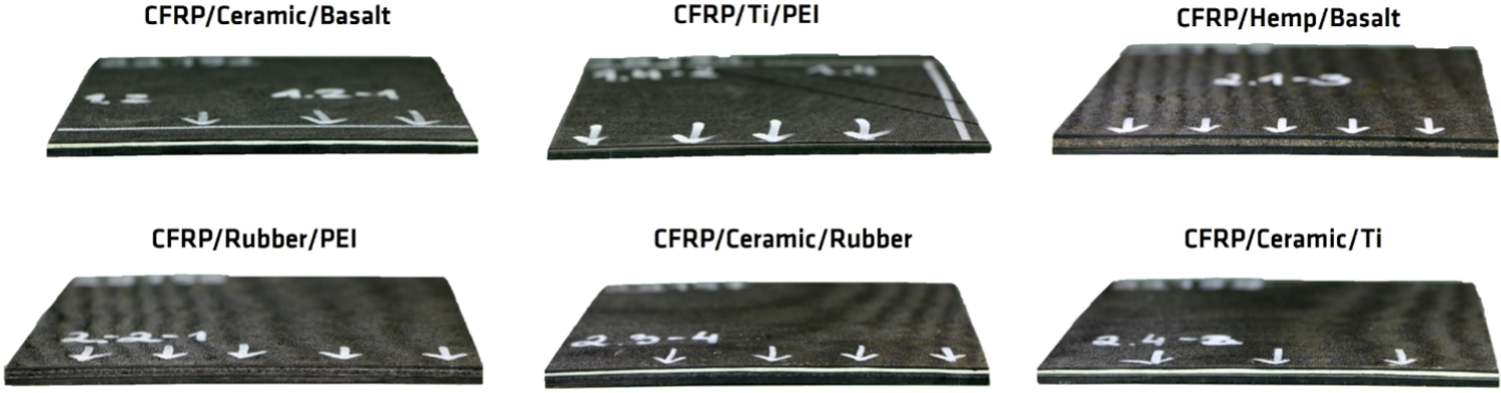
Six laminate systems with protective interlayers.

The specimens were cut by water jet cutting (Ridder HWE P10–10, Fa. Ridder; Germany). The GMA abrasive mesh 120 was fed at a flow rate of 200 g/min. The stand-off distance was 3 mm, and the feed rate was 600 mm/min. Because laminates with Ti foil tend to delaminate during cutting, the working pressure was set higher for these composites to 2800 bar; for all others, it was 2000 bar. The specimen prepared for the fire resistance test was 120 mm × 120 mm in size. The thickness of the specimens varied from 3.6 to 5.6 mm. The 6 different systems of CFRP laminates were prepared with varying protective interlayers. All of the configurations are shown in Table 2. Photographs of the specimens are presented in Figure 2. The reference specimen was a quasi-isotropic laminate consisting of 24 CF layers: [±/90/–/0/+/90/0/–/90/+/0]s.

**Table 2:** 6 CFRP Systems with Protective Interlayers

	1st protective interlayer	2nd protective interlayer	thickness/mm
1.	ceramic composite WHIPOX	basalt-fiber-woven mat	4.3
2.	titanium foil	PEI foil	3.6
3.	hemp-fiber mat	basalt-fiber-woven mat	5.6
4.	rubber mat “Pyrostat”	PEI foil	4.7
5.	ceramic composite WHIPOX	rubber mat “Pyrostat”	5.2
6.	ceramic composite WHIPOX	titanium foil	4.7

### 2.3 Bench-Scale Fire Resistance Test

The fire resistance test was carried out in a bench-scale setup designed at Bundesanstalt für Materialforschung und -prüfung (BAM, Berlin).^6^ Adopted from Gibson,^11,15−17,43^ the setup consists of a compression device, hydraulic pump, and propane burner. The hydraulic machine was connected to a power supply and an OM-USB-TC-AI data acquisition module (OMEGA Engineering GmbH, Germany), which transferred the data to a computer where they were displayed and analyzed using TracerDAQ software. The sample was fixed at the bottom and guided along the side edges. The load was applied by a hydraulic machine to the compression device. Two interchangeable pressure cylinders were used to adjust the compression force: Enerpac RC-106 with a maximum compressive force of 101.5 kN for the fire resistance test and Enerpac RC-256 cylinder with a maximum compressive force of 230 kN to estimate the ultimate failure load of the specimens. A gas burner with a nozzle diameter of 60 mm was connected to an EL-FLOW metal sealed gas mass flow meter (Bronkhorst High-Tech B. V., Netherlands) to control a constant gas flow. The integrated water-cooling system was attached to a compression device to avoid thermal expansion during the fire test. Before the fire resistance test, the proper heat flux (180 kW/m^2^) and temperature (about 1020 °C) of the flame was adjusted by varying the gas flow. These parameters are in accordance with flame application for fire tests in the aviation sector, e.g., 14 CFR 25.856 Appendix F Part VII;2003. The distance between the burner and specimen was 27.5 cm. Heat flux measurements were conducted using a water-cooled Meditherm (Gardon gauge type, Serial #184881) fixed in the ceramic reference plate (Fiberfrax Duraboard, thickness 10 mm). Temperature measurement was performed by a thermocouple located next to the heat flux meter. During calibration and fire testing, the compression device was covered with glass wool.

#### 2.3.1 Static Load Test

The ultimate failure load was determined in a static load test at room temperature (without the application of fire). A slowly increasing compressive load was applied to the specimen until failure. The failure point is observed on the load versus time graph displayed by the TracerDAQ software. For the pure CFRP shell specimen, the ultimate failure load was ∼81 ± 4.44 kN. Additionally, the static load test was also conducted for two systems with protective interlayers: CFRP/Ceramic/Basalt and CFRP/Kenaf/Basalt. The laminates with protective interlayers achieved the same results, showing that the integration of additional interlayers into the CFRP laminate does not negatively affect the mechanical properties. In addition, the static load test was carried out on two specimens with minor edge defects after cutting. For these specimens, a clear reduction in failure load was observed, implying that laminate preparation is a pivotal, critical step that directly impacts the ultimate properties of the material. However, additional testing (e.g., flexural tests, impact testing, fatigue testing) would be needed to observe the influence of additional interlayers on mechanical properties of laminates. The results are presented in Table 3.

**Table 3:** Ultimate Failure Load of CFRP Specimens

	ultimate failure load/kN	
reference CFRP	81 ± 4.44	
CFRP with protective interlayers	79.02	85.99
CFRP with protective interlayers and defects	44.16	59.73

#### 2.3.2 Fire Resistance Test

**Figure 3:**
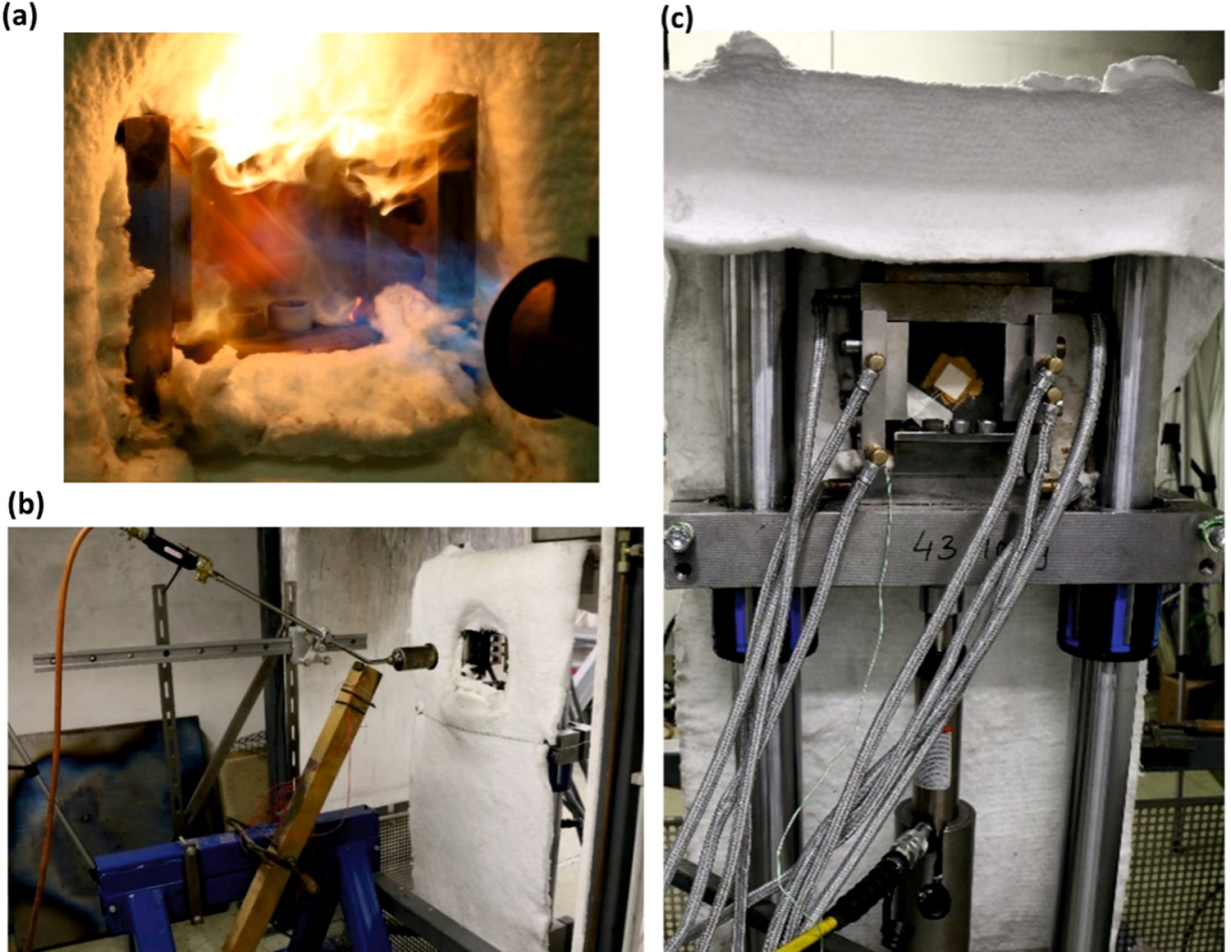
(a) Fire resistance test in bench scale. (b) Front view of the setup protected with Kaowool from direct fire. (c) Back view of the setup, where thermocouple is attached to the specimen.

Ten percent of the ultimate failure load (8 kN) was applied to the specimen beforehand and held constant throughout the fire test. After the burner was heated for 30 s, fire was applied directly to the one side of the specimen, and the time to failure was measured. Failure was visible as a sharp drop in the load-time graph displayed by TracerDAQ software. The temperature at the back of the specimen was measured using a type K thermocouple bonded by a ceramic adhesive, as shown in Figure 3c. Figure 3 shows the bench-scale fire resistance setup.

### 2.4 Analysis of Failures Using an X-ray Computed Tomography (XCT) System

**Figure 4:**
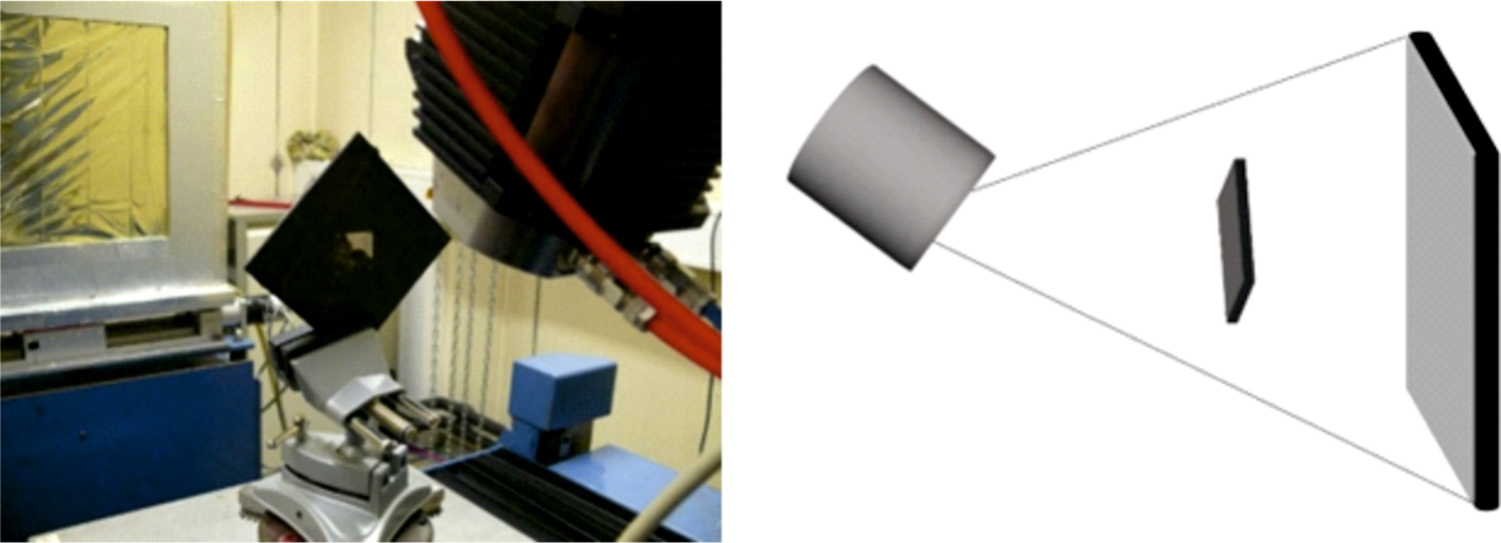
Photograph of the used CT system with the sample clamped on the rotating table (left) and schematic of the X-ray CT measurement principle (right).

X-ray computed tomography (XCT) imaging was conducted by using a system developed by researchers at BAM together with the company Sauerwein Systemtechnik (now RayScan Technologies, Germany). The X-ray source was a 225 kV microfocus X-ray tube XWT-225-SE (X-ray WorX GmbH, Germany) with a focal spot size of 6 μm. The 2048 × 2048 pixel (200 μm pitch) amorphous silicon detector panel XRD 1620 (PerkinElmer, Germany) converted X-rays to visible light via a scintillation layer. A photograph of the setup is shown in Figure 4. The specimen was imaged with a resolution of 27.8 μm (voxel size). Three thousand images were taken during the 360° rotation of the object. The evaluation program is VGStudio MAX 2023.1 from Volume Graphics GmbH (Heidelberg, Germany).

## 3 Results and Discussion

### 3.1 Fire Stability Test of CFRP Laminates with Protective Interlayers

**Figure 5:**
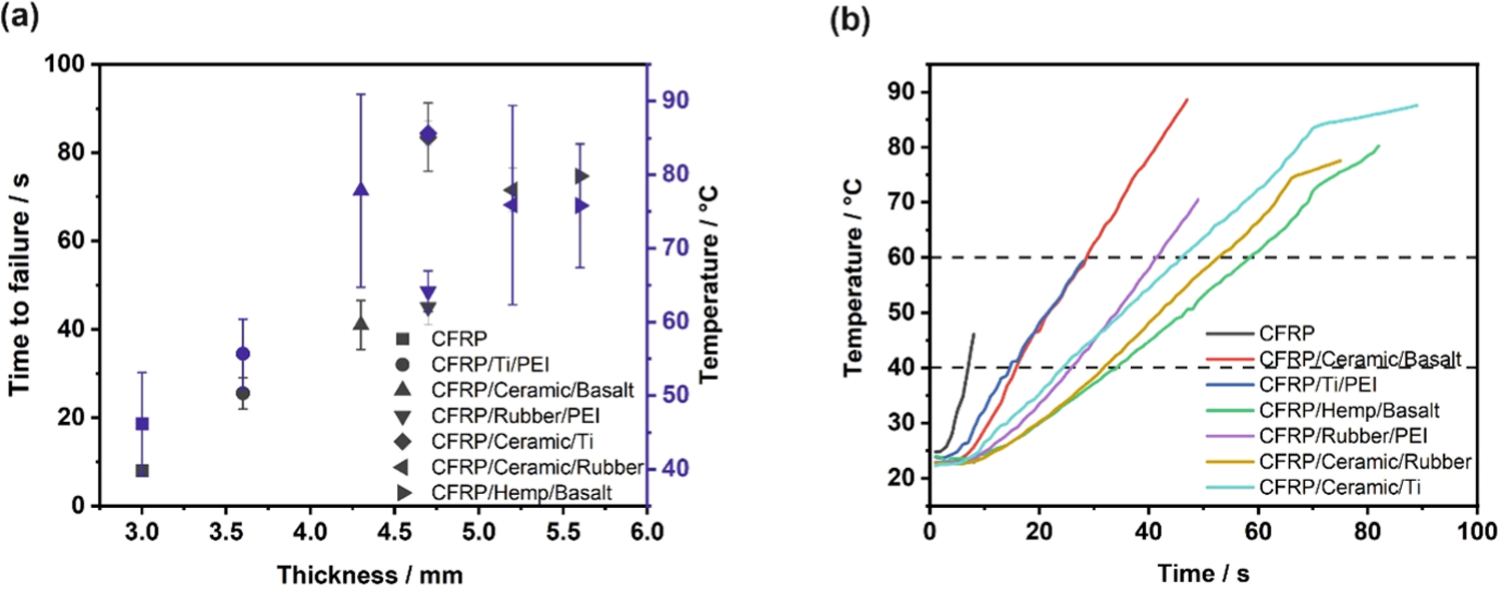
(a) Time to failure and temperature at failure as a function of thickness; (b) temperature profiles of CFRP laminates.

**Figure 6:**
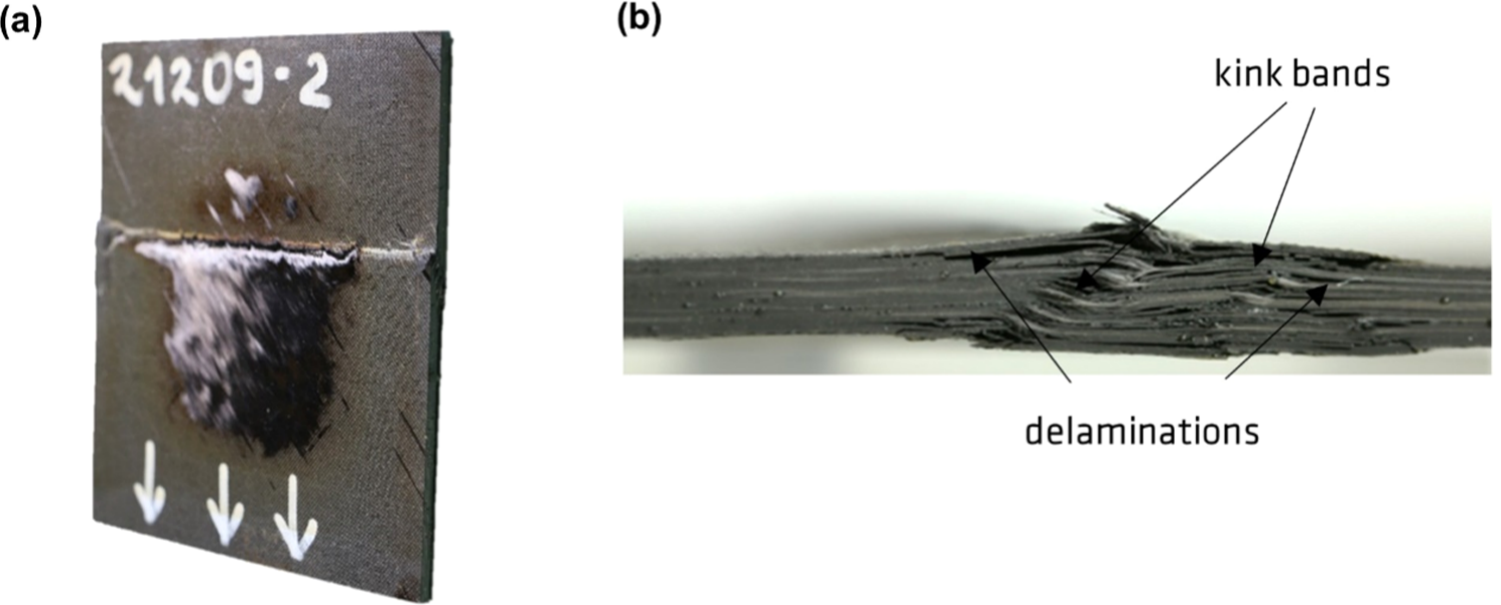
Residues of pure CFRP. (a) View from side; (b) view from the top.

Thickness is known to be a critical parameter exerting a significant influence on fire resistance. As studied by Timme,^18^ the critical buckling load increases significantly with increasing thickness of CFRP shell specimens, as observed particularly in the bench-scale test. Although all specimens consist of the same lay-up of 24 carbon fiber layers, each system has different protective interlayers of varying thickness, resulting in different final specimen thicknesses. Figure 5a shows the influence of thickness on the time to failure and temperature at failure. Besides the CFRP/Ceramic/Ti laminate, the specimens showed a linear increase in time to failure with increasing thickness. However, CFRP/Ceramic/Ti achieved a time to failure almost double that of CFRP/Rubber/PEI with this same thickness. This observation implies that the fire resistance of the specimen does not depend solely upon its thickness but also on the distinctive properties inherent to each individual protective interlayer. Table 4 shows the recorded failure data from the bench-scale fire stability test. CFRP/Ceramic/Ti failed after 84 s. The ceramic layer is a nonbrittle material developed for high-temperature applications; thus its combination with CFRP and titanium foil brings highly effective results. Two pure CFRP reference specimens failed after 8 s. Without any form of protection from direct fire, the epoxy matrix quickly approached its glass transition temperature (∼177 °C) and decomposed. This resulted in heat transfer to the inner layers of the CFRP composite and immediate failure. Figure 6a shows the pure CFRP specimen after the fire stability test. The epoxy matrix is completely burnt out, and a round area with pure carbon fibers was formed, indicating the place where the specimen was exposed to fire. The horizontal line in the middle is attributed to the formation of large wrinkling, called buckling. This is the characteristic failure behavior for all CFRP laminates. The failure mechanism primarily involves the propagation of cracks that align parallel to the direction of the load. In Figure 6b, the side view of CFRP residue is shown, illustrating the different failure modes: delamination and kink bands. All specimens with integrated interlayers achieved a longer time to failure (up to 10 times longer) and significantly improved fire stability. The protective interlayers with lower thermal conductivity provided thermal insulation and reduced heat transmission. As Schartel^43^ and Hörold^43,44^ previously observed, upon exposure to fire, the surface of laminates undergoes rapid heating, surpassing the decomposition temperature. Subsequently, heat propagates inward, wherein the protective interlayers effectively diminish the heat conductivity. The decomposition of the laminate through thickness slows down, reducing the thermal conductivity and average temperature through thickness, and delays the softening of the matrix as the thickness of the laminate increases.^45,46^ As the specimen withstands the fire resistance test for a longer time, a higher temperature is reached at the back of the specimen in the moment of failure.

**Table 4:** Results of the Bench-Scale Fire Resistance Test

				time to reach temperature/s		
	time to failure/s	temperature at failure/°C	heating rate: slope/°C/s	40 °C	60 °C	thickness/mm
CFRP	8	46.1 ± 7	3.7	7 ± 1	-	3.1
CFRP/ceramic/basalt	41 ± 5	77.8 ± 13	1.6	16 ± 2	29 ± 3	4.3
CFRP/Ti/PEI	26 ± 3	55.7 ± 5	1.5	15	-	3.6
CFRP/hemp/basalt	75 ± 7	75.8 ± 8	0.8	34 ± 6	58 ± 6	5.6
CFRP/rubber/PEI	45 ± 4	64.2 ± 3	1.3	26 ± 1	42 ± 1	4.7
CFRP/ceramic/rubber	72 ± 5	75.9 ± 13	0.9	32	54 ± 7	5.2
CFRP/ceramic/Ti	84 ± 8	85.6 ± 1	0.9	24 ± 1	46 ± 4	4.7

Figure 5b shows the temperature versus time at the unexposed face of the laminates. The noticeable delay of several seconds before the temperature begins to rise indicates that protective interlayers effectively delayed the transmission of heat through the laminate structure. The slope of the temperature–time curve represents the heating rates of each system, which are shown in Table 4. The laminates with protective interlayers show a great reduction in heating rates by as much as 56–78% in comparison with the pure CFRP. There is a slight shift in the time before the temperature begins to rise in laminates with protective interlayers. For the specimens with the longest time to failure (CFRP/Hemp/Basalt, CFRP/Ceramic/Rubber, and CFRP/Ceramic/Ti), which reached higher temperatures on the back surface, a specific “bending point” before failure was visible. The thermal conductivity in the thickness direction of these laminates was reduced by volatile gases, which were released at higher temperatures.^15,47^ The dashed lines indicate the time before the laminates reached 40 and 60 °C; the results are presented in Table 4. As reported by Hume,^48^ thicker specimens have a higher heat capacity and are therefore characterized by a longer time to reach a resin decomposition temperature.

### 3.2 Position of the Protective Layer vs Its Function

A critical preliminary phase preceding the manufacturing of laminates involves the strategic determination of positioning and material selection for the protective interlayers. The functions of each protective interlayer are designed theoretically to be studied later in the fire resistance test. The application of the same material in two different positions in two systems allows the investigation of how the positioning of the layer within the lay-up configuration influences the result. The ceramic composite was applied as a first protective interlayer in three distinct systems: CFRP/Ceramic/Basalt, CFRP/Ceramic/Rubber, and CFRP/Ceramic/Ti provide an opportunity to juxtapose the materials used as a second protective interlayer. Although basalt fibers have a high operating temperature limit (∼700 °C), the laminate with a basalt mat is the thinnest and exhibits the shortest time to failure: only 41 s (Table 4). Interestingly, despite this shorter duration to failure, the temperature measured at the back of the CFRP/Ceramic/Basalt laminate was registered at 77.8 °C, mirroring the temperature recorded for the laminate with a rubber mat, which was exposed to fire for an additional 30 s. This phenomenon is attributed to the reduced rate of heat transfer through the rubber mat, as indicated by a 44% reduction in the heating rate observed in the CFRP/Ceramic/Rubber laminate. Although the CFRP/Ceramic/Rubber specimen had the greatest thickness, it notably achieved a longer time to failure than the CFRP/Ceramic/Basalt laminate. However, the longest time to failure was accomplished by the CFRP/Ceramic/Ti system, lasting 83.5 s, merely 0.4 mm thicker than the laminate incorporating the basalt mat. Consequently, it is evident that the titanium foil provides enhanced structural integrity compared with other protective interlayers, demonstrating superior performance even with a marginal difference in thickness.

**Figure 7:**
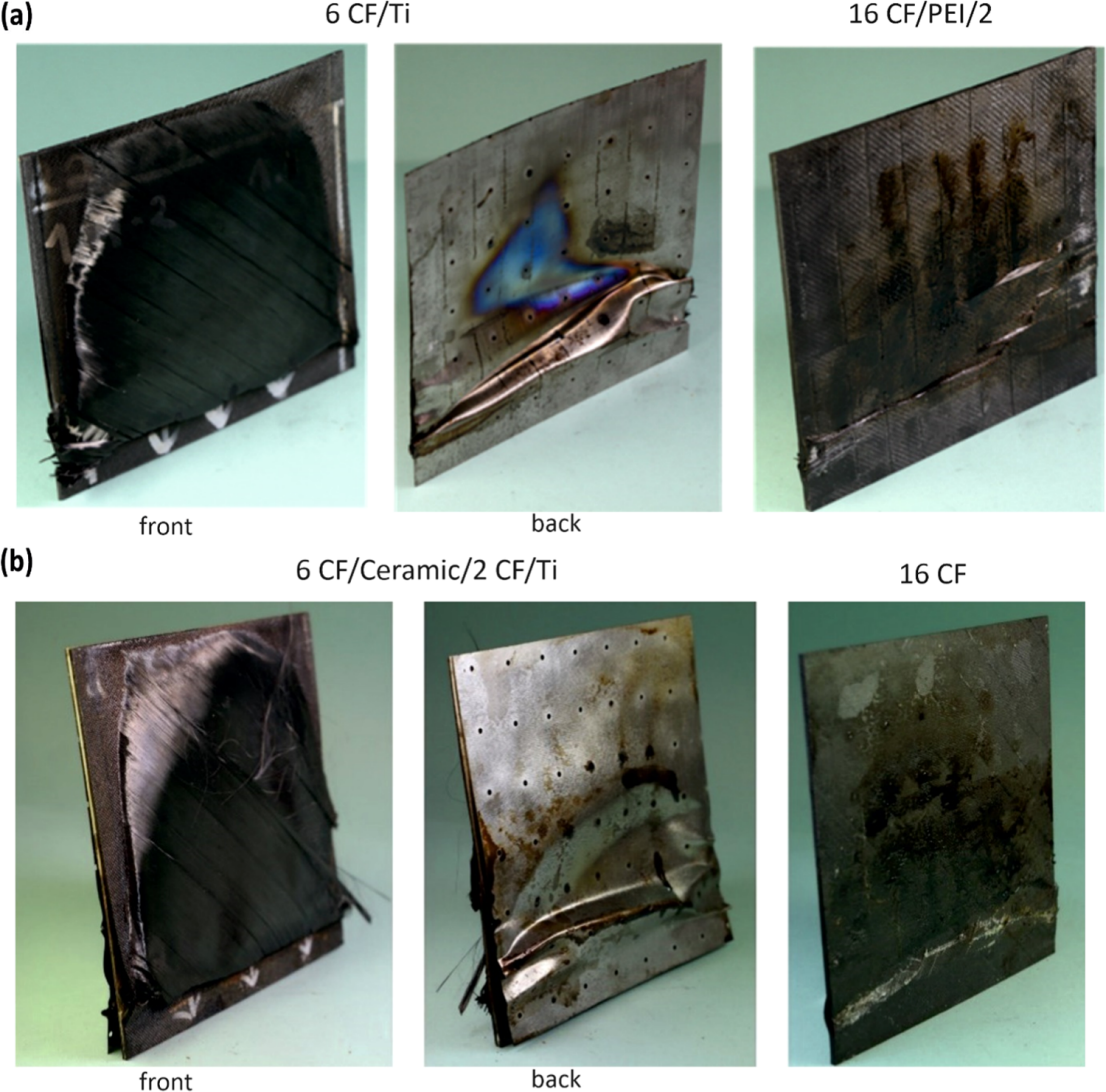
Photos of disintegrated residues: (a) CFRP/Ti/PEI and (b) CFRP/Ceramic/Ti laminates.

The laminates with the Ti foil experienced delamination and separated into two distinct parts upon failure. Figure 7a shows a photograph of titanium foil applied as a first protective interlayer in CFRP/Ti/PEI, situated approximately 6 CF layers (∼0.76 mm) away from the flame. The blue area represents the zone of heat transfer to the inner laminate. Figure 7b shows the remainder of this laminate, consisting of 2 CF layers, PEI foil, and 16 CF layers. The heat transfer resulted in further epoxy resin decomposition, as evidenced by smoldering on the surface. As reported by Parlevliet,^27^ the PEI foil is able to create insulating gaps with gases within the CFRP laminate, effectively preventing rapid heat transfer to the deeper layers. However, the epoxy resin matrix burned out from the initial layer, and the volatile components were not trapped for long, resulting in the loss of the barrier layer. In addition, the Ti foil in the CFRP/Ceramic/Ti configuration (Figure 7b), which was positioned as the second protective interlayer, had no transfer area, resulting in increased distance to the flame of approximately 1.51 mm due to the first protective layer and 8 CF layers (in two parts in the lay-up). This specimen separated into two parts upon failure: one consisting of 6 CF layers with a ceramic layer and 2 CF layers and the other comprising Ti foil with 16 CF layers. The ceramic layer, which served as the initial protective interlayer, effectively acted as a barrier, preventing significant heat transfer to the internal laminate layers. WHIPOX ceramic composites are characterized by high porosity,^21,23^ which could improve bonding with carbon fibers and thus enhance the structural integrity of the laminate. In addition, the CFRP/Ti/PEI configuration exhibited a time to failure that is only one-third as long as that of the CFRP/Ceramic/Ti laminate. This result underscores the critical role of the first protective interlayer as the primary insulation barrier, which limits rapid heat transfer to the deeper layers within the laminate structure. Consequently, selecting the appropriate material for the initial barrier layer stands out as a crucial design aspect significantly influencing the fire-resistant performance of the laminate.

**Figure 8:**
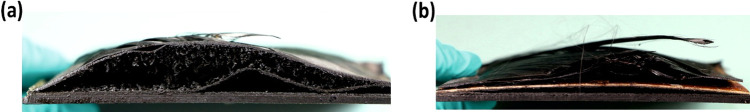
Top-view images of residues of (a) CFRP/Rubber/PEI and (b) CFRP/Ceramic/Rubber.

A similar relationship was observed in laminates featuring the positioning of a rubber mat as both the first and second protective interlayers. The “Pyrostat” rubber mat begins to expand when it is heated above 200 °C and forms a fire-resistant and smoke-proof barrier. Figure 8 illustrates top-view images of laminate residues consisting of a rubber mat. In the case of CFRP/Rubber/PEI (Figure 8a), heating caused the expandable graphite within the rubber mat to expand, forming a thick, intumescent barrier against fire. However, this notable phenomenon was absent in the residue of CFRP/Ceramic/Rubber (Figure 8b), where the rubber mat served as the second protective interlayer. This indicates that the ceramic layer effectively protected the laminate, impeding sufficient heat transfer to the inner sections of the specimen before failure occurred. Furthermore, the superior performance of the ceramic layer is reflected in the results of the fire stability test, with the CFRP/Rubber/PEI laminate failing after 45 s, whereas the CFRP/Ceramic/Rubber survived for 71.5 s.

Recently there has been increased interest in exploring the potential of natural fibers as alternatives in composite materials.^49^ The fire resistance evaluation of the laminate composed of CFRP, hemp fibers, and basalt mat showed fire protective capabilities comparable to commercially available synthetic interlayers. Results of the fire resistance test are presented in Table 4. Hemp fibers demonstrated excellent thermal isolation properties within the laminate structure and failed after 75 s, which is comparable to the laminate with a ceramic layer and rubber mat (CFRP/Ceramic/Rubber), which failed after 72 s. Notably, the laminates with hemp fibers, which were the thickest laminates, experienced a reduced heating rate compared to the other laminate systems, showcasing the lowest rate of temperature elevation during the fire resistance test. It has been already observed by Dahal^50^ and Sarkar^51^ that hemp fibers have very good insulation properties; thus, they are often used in buildings as an insulating material. Overall, the experimental outcomes demonstrate that integrating the CFRP laminate with natural fibers like hemp and basalt mat presents a compelling prospect for use in scenarios demanding enhanced fire resistance. This combination shows promise in providing a sustainable and efficient substitute for traditional synthetic materials, potentially catering to applications where robust fire protection is essential.

Basalt mat was also applied as a second protective interlayer in CFRP/Ceramic/Basalt. Although the ceramic composite WHIPOX has very high temperature stability (up to 1300 °C), the time to failure of CFRP/Ceramic/Basalt was almost half the length, 41 s, and the heating rate was twice as high. Since the CFRP/Hemp/Basalt laminate is 1.3 mm thicker, the primary factor influencing these results was the thickness. Surprisingly, however, the failure temperature of the laminate with ceramic composite was as high as that of the laminate with hemp fibers. This unexpected result can be attributed to the significantly higher thermal conductivity of the WHIPOX composite layer—2.7 W/m·K^52^—which is 70 times higher than the thermal conductivity of hemp fibers at 0.039 W/m·K.^50^ The application of this same material as a second protective interlayer allows the materials used as a first protective interlayer to be compared.

This concept was also investigated in laminates where PEI was applied as a second interlayer: in CFRP/Ti/PEI and CFRP/Rubber/PEI. However, the results demonstrated notable consistency and followed a linear trend that depended mostly on the thickness of the specimens. Since the rubber mat is thicker than titanium foil, the total thickness of the specimen increased and affected the fire stability of the laminate. The time to failure of the CFRP/Rubber/PEI laminate was 45 s, which is 1.7 times longer than the time to failure of CFRP/Ti/PEI—26 s. Since the time to failure was extended, the duration of exposure to fire was also longer; thus, there was a corresponding increase in the temperature at which the laminate with rubber mat failed. However, the heating rate of this laminate was reduced only slightly.

### 3.3 Failure Analysis

**Figure 9:**
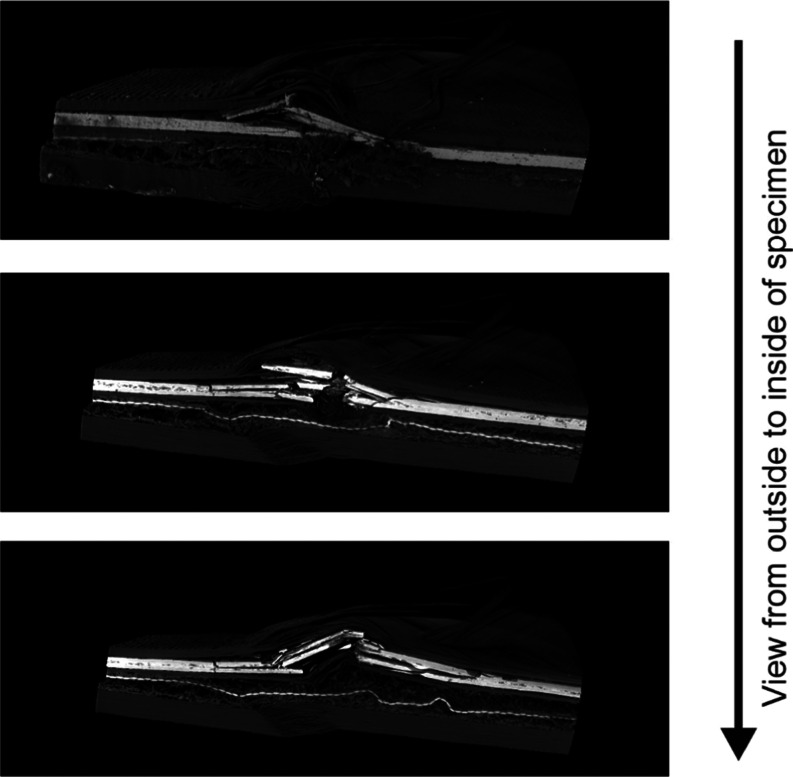
X-ray CT Image of CFRP/Ceramic/Rubber.

X-ray CT analysis enables the assessment of the various modes of compression failure within the laminate. The study was carried out at the CFRP/Ceramic/Rubber laminate, which showed the greatest variety of failures. Figure 9 presents three images taken at different points across the specimen: outside and two parallel cross sections. All images show the kink band formation in the front part of the laminate, consisting of six CF layers, which was directly exposed to fire. This failure mode is characteristic at temperatures above the glass transition temperature when polymer matrix softens. Multiple translaminar fractures and delaminations of ceramic layer indicate that the stiffness and brittleness of the material was higher than that for carbon fibers. Furthermore, the kink bands and delaminations were visible in the two carbon fiber layers, which were placed between protective interlayers. The activated expandable graphite, which was embedded in the rubber mat, is visible in the external side view of the laminate. The flame hit in the middle of the specimen and spread outward, hence the temperature there was able to increase locally. However, this phenomenon did not occur in the following two cross-sectional images of the residue. The ceramic layer served as an efficient protective and insulating barrier for the rest of the specimen, effectively preventing the expandable graphite from reaching its activation temperature (270 °C). Due to the substantial thickness and inherent softness of the rubber mat, only minor microbucklings occurred as failures. In the thickest segment of the laminate, consisting of 16 layers of carbon fiber, characteristic buckling delamination behavior was observed.

## 4 Conclusions

In this research, the fire resistance of CFRP laminates with integrated protective interlayers was investigated at the bench-scale. The results showed that incorporation of protective interlayers led to a notable improvement in the fire stability of CFRP laminates. Protective interlayers significantly delayed heat transfer throughout the laminate structure, thereby extending the time of softening, which resulted in reduced heating rates. Moreover, the specific configuration involving the application of protective interlayers with 8 carbon layers in the front (in two sections: 2 CF and 6 CF) enables the subsequent section (comprising 16 carbon fiber layers) to be shielded from direct fire and remains for an extended duration to carry the load. The integration of two different protective interlayers made it possible to investigate their cooperative effects, which include reduced heat conductivity. Testing different systems in which some share an identical initial or second interlayer offers the opportunity to examine both the efficacy of the protective interlayer and the synergistic interactions between protective interlayers. This comparison indicated that fire stability is determined not solely by the thickness of the specimens but also by the specific properties of each individual interlayer. Moreover, the outcomes indicated that selecting an appropriate material for the protective interlayer was a critical design consideration that directly influenced the fire resistance performance. As observed, the first interlayer plays a critical role as the first insulating barrier. It stops heat transfer to the next parts of the laminate. Therefore, the thermal conductivity and thickness of protective interlayers are important parameters that influence the fire resistance of laminates. Among the various systems investigated, the study revealed that the combination of the ceramic layer with titanium yielded the most optimal results in terms of fire stability. The ceramic composite WHIPOX also has high thermal stability and is characterized by high porosity, improving its connection to carbon fiber layers and enhancing the structural integrity of the laminate. The comparison of different laminates indicates Ti foil’s efficacy in preserving the structural integrity when exposed to fire, suggesting its potential for enhancing fire stability in composite materials. The CFRP laminate with hemp and basalt mat presents an excellent possibility to use natural fibers to create composites that exhibit fire-resistant properties similar to commercially available synthetic products. Due to the different stiffnesses of the protective interlayers, a variety of compression failure modes has been observed in laminates.

## Supporting Information Available

Additional experimental details, properties of materials, and photograph of CFRP specimen after static load test (PDF)
