# Supplementary material for: Sacrifice Few to Save Many: Fire Protective Interlayers in Carbon-Fiber-Reinforced Laminates
Source: ACS Omega. 2024 May 22;9(22):23703–12. doi: 10.1021/acsomega.4c01408 (PMC11154947; doi:10.1021/acsomega.4c01408)
Supplement: Supplementary file 1 — ao4c01408_si_001.pdf [file ao4c01408_si_001.pdf]

SUPPORTING INFORMATION

Sacrifice few to save many: Fire protective interlayers in carbon-fibre-reinforced laminates

*Weronika Tabaka, Dietmar Meinel, Bernhard Schartel**

Bundesanstalt für Materialforschung und –prüfung (BAM), Unter den Eichen 87, 12205 Berlin, Germany

Corresponding Author

*Bernhard Schartel, bernhard.schartel@bam.de

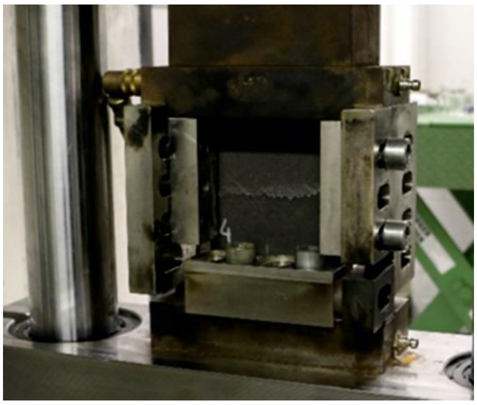

Figure S.1. CFRP specimen in compression device after static load test

Table S.1. Properties of EPIKOTE Resin MGS RIMR 935 and EPIKURE Curing Agent MGS RIMH 937

		Resin RIMR 935	Hardener RIMH 937
**Density**	g/cm^3	1.14-1.20	0.92-0.96
**Viscosity**	mPas	400-800	30-100
**Epoxy eqivalent**	g/equivalent	155-165	-
**Epoxy value**	Eqivalent/100g	0.61-0.64	-
**Amine value**	mg KOH/g	-	450-500

Table S.2. Properties of protective interlayers.

Specimen name	Trade name	Company	Thickness / mm	Surface weight / g/m^2	Density / g/m^3	Thermal Conductivity / W/m·K
Ceramic composite	WHIPOX	WPX Faserkeramik GmbH	0.5	-	2.9	2.7
Titanium foil	Grade 2	ATI Flat Rolled Products GmbH	0.125	-	4.511	22.5
PEI foil	Ajedium Ultra 1000	Solvay	0.125	-	1.28	0.220
Basalt-fibre woven mat	-	Incotelogy LTD	0.1	210	-	0.031-0.038
"Pyrostat" rubber mat	Pyrostat Uni	G+H Isolierung	1.1	1200	-	1.056
Hemp-fibre mat	-	Polyvlies Franz Beyer	2.2 mm	700	-	0.039

Thermal Conductivity of "Pyrostat" rubber mat was measured at BAM with TPS 1500 from Hot Disk (Gothenburg, Sweden). All other properties have been specified in the data sheet of the materials.
